# Exploring causal correlations of inflammatory biomarkers in idiopathic normal-pressure hydrocephalus: insights from bidirectional Mendelian randomization analysis

**DOI:** 10.3389/fnagi.2024.1412434

**Published:** 2024-06-21

**Authors:** Jianglong Lu, Xianpeng Wang, Fanjie Xu, Changjun Rao, Yuhang Guo, Zhipeng Su, Siyan Chen, Qun Li

**Affiliations:** ^1^Department of Neurosurgery, First Affiliated Hospital of Wenzhou Medical University, Wenzhou, China; ^2^Beijing Neurosurgical Institute, Beijing Tiantan Hospital, Capital Medical University, Beijing, China; ^3^Department of Neurology, First Affiliated Hospital of Wenzhou Medical University, Wenzhou, China

**Keywords:** idiopathic normal-pressure hydrocephalus, biomarkers, inflammation, Mendelian randomization, meta-analysis

## Abstract

**Background and objective:**

Neuroinflammatory processes have been identified as playing a crucial role in the pathophysiology of various neurodegenerative diseases, including idiopathic normal-pressure hydrocephalus (iNPH). iNPH, defined as a common disease of cognitive impairment in older adults, poses major challenges for therapeutic interventions owing to the stringent methodological requirements of relevant studies, clinical heterogeneity, unclear etiology, and uncertain diagnostic criteria. This study aims to assess the relationship between circulating inflammatory biomarkers and iNPH risk using bidirectional two-sample Mendelian randomization (MR) combined with meta-analysis.

**Methods:**

In our bidirectional MR study, genetic data from a genome-wide association study (GWAS) involving 1,456 iNPH cases and 409,726 controls of European ancestry were employed. Single-nucleotide polymorphisms (SNPs) associated with exposures served as instrumental variables for estimating the causal relationships between iNPH and 132 types of circulating inflammatory biomarkers from corresponding GWAS data. Causal associations were primarily examined using the inverse variance-weighted method, supplemented by MR-Egger, weighted median, simple mode, and weighted mode analyses. In the results, heterogeneity was assessed using the Cochran *Q* test. Horizontal pleiotropy was evaluated through the MR-Egger intercept test and the MR pleiotropy residual sum and outliers test. Sensitivity analysis was conducted through leave-one-out analysis. Reverse MR analyses were performed to mitigate bias from reverse causality. Meta-analyses of identical inflammatory biomarkers from both data sources strengthened the findings.

**Results:**

Results indicated a genetically predicted association between Interleukin-16 (IL-16) [OR: 1.228, 95% CI: 1.049–1.439, *p* = 0.011], TNF-related apoptosis ligand (TRAIL) [OR: 1.111, 95% CI: 1.019–1.210, *p* = 0.017] and Urokinase-type plasminogen activator (uPA) [OR: 1.303, 95% CI: 1.025–1.658, *p* = 0.031] and the risk of iNPH. Additionally, changes in human Glial cell line-derived neurotrophic factor (hGDNF) [OR: 1.044, 95% CI: 1.006–1.084, *p* = 0.023], Matrix metalloproteinase-1 (MMP-1) [OR: 1.058, 95% CI: 1.020, 1.098, *p* = 0.003] and Interleukin-12p70 (IL-12p70) [OR: 0.897, 95% CI: 0.946–0.997, *p* = 0.037] levels were identified as possible consequences of iNPH.

**Conclusion:**

Our MR study of inflammatory biomarkers and iNPH, indicated that IL-16, TRAIL, and uPA contribute to iNPH pathogenesis. Furthermore, iNPH may influence the expression of hGDNF, MMP-1, and IL-12p70. Therefore, targeting specific inflammatory biomarkers could be promising strategy for future iNPH treatment and prevention.

## 1 Introduction

Inflammation has long been associated with neurodegeneration, and systemic levels of proinflammatory cytokines affect neural circuit plasticity in response to external stimuli (Mukandala et al., 2016). Idiopathic Normal Pressure Hydrocephalus (iNPH), as a common neurodegenerative disease, has the following clinical signs: (a) impaired gait or balance, cognitive disturbance, or impaired urinary incontinence, Symptoms have been insidious for at least 3 months or longer; (b) cranial imaging showed ventricle enlargement with an Evan’s index >0.3; (c) Lumbar CSF opening pressure less than 18 mmHg (Relkin et al., 2005). iNPH is estimated to affect 10–22 individuals per 100,000, with 1.30 and 5.9% of those affected aged ≥65 and ≥80 years, respectively (Martín-Láez et al., 2015). The precise mechanisms underlying iNPH remain somewhat elusive (Nassar and Lippa, 2016; Bonney et al., 2022), although ventriculomegaly resulting from cerebrospinal fluid (CSF) dynamics may initiate a vicious cycle of neurological damage in those with the disorder. Pathophysiological factors, such as hypoperfusion, glymphatic impairment, metabolic disturbance, astrogliosis, neuroinflammation, and blood–brain barrier (BBB) disruption contribute to both white and gray matter lesions, ultimately manifesting in various iNPH symptoms (Wang et al., 2020).

Until now, extensive researchers have explored the association between iNPH and inflammatory biomarkers in an attempt to identify reliable biomarkers (Tarnaris et al., 2006, 2009; Braun et al., 2023). Several cerebrospinal fluid proteins are potentially important in iNPH or Alzheimer’s disease. Levels of total tau (t-tau), phosphorylated-tau (p-tau), and amyloid-*β42* (A*β42*) are often altered in related neurodegenerative diseases (Leinonen et al., 2011). CSF levels of the proinflammatory factors Tumor necrosis factor alpha (TNFα) have exhibited marked changes before and after shunt surgery in patients with iNPH (Tarkowski et al., 2003). Observational researches have indicated the elevation of IL-6, IL-1β, and LRG levels in the CSF of patients with iNPH compared to healthy controls (Czubowicz et al., 2017; Lolansen et al., 2021). However, due to the methodological biases inherent in observational studies, as well as small sample sizes, no definitive conclusions have been drawn regarding the causal relationship between these biomarkers and iNPH risk (Tarnaris et al., 2009). However, effective for diagnostic, prognostic, and therapeutic response biomarkers are still largely lacking, especially blood-based biomarkers.

To date, Mendelian randomization (MR) has not been used to investigate the causal relationship between circulating inflammatory biomarkers and iNPH. To fill this gap, we combined MR with meta-analysis based on genome-wide association study (GWAS) data to investigate the potential relationship between these biomarkers and iNPH risk. MR is an effective method for evaluating associations between exposure and disease, using genetic variation as an instrumental variable (Bowden and Holmes, 2019). Given random allocation of inherited variants during gamete formation, MR analysis helps mitigate potential confounding effects and reverse causality (Smith and Ebrahim, 2003). Confirmation of circulating inflammatory biomarkers’ involvement in iNPH pathogenesis would not only advance the identification of potential drug targets for iNPH treatment, but also hold diagnostic and prognostic value for iNPH.

## 2 Materials and methods

### 2.1 Study design

**Figure 1:**
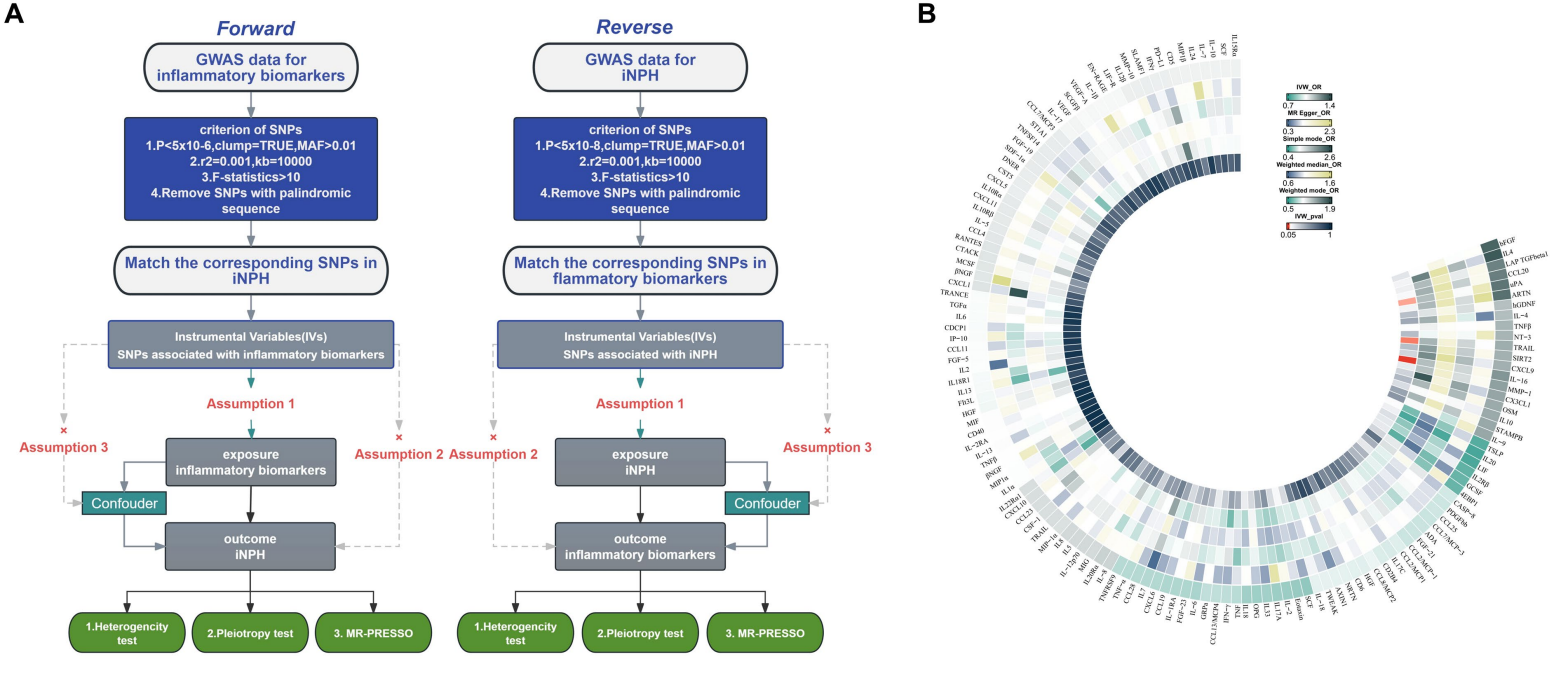
**(A)** Flowchart of the Mendelian randomization study of the causal association between 132 inflammatory biomarkers and iNPH. **(B)** Primary analysis of the association of 132 inflammatory biomarkers and iNPH. MAF, Minor allele frequency; SNP, Single-nucleotide polymorphism; iNPH, idiopathic Normal pressure hydrocephalus; IV, instrumental variable; IVW, Inverse variance weighted; OR, Odds Ratio.

The overview of the study design is shown in Figure 1. We employed a two-sample MR design to investigate causal relationships between 132 circulating inflammatory biomarkers and iNPH. Using publicly available GWAS data, which were approved by relevant institutional review boards, ensured that no additional informed consent or ethical approval was required. MR analysis should adhere to three core assumptions: (1) a strongly association between the instrument and exposure; (2) an absence of confounding variables influencing both the risk factor and the outcome, and no association between these variables and the genetic instrument linked to the risk factor and outcome; and (3) the instrument has no direct effect on the outcome except from through exposure (Sanderson et al., 2022).

### 2.2 Data sources

The datasets used in the MR analysis were sourced from publicly available summarized GWAS data. Regarding inflammatory biomarkers, we aggregated data from the two largest datasets available for circulating inflammatory cytokines, allowing us to draw more reliable conclusions. Among these, 41 inflammatory cytokines GWAS originated from a study on genetic associations of circulating inflammatory cytokines and growth factors in 8,293 Finnish individuals (Ahola-Olli et al., 2017), including two study cohorts, the Cardiovascular Risk in Young Finns Study (mean age male: 37.4 years; Women: 37.5 years) and the FINRISK study (men mean age FINRISK1997:48.3; Female: 47.3, FINRISK2002 Male: 60.4; Female: 60.1). GWAS data for these 41 inflammatory cytokines can be found in the IEU Open GWAS project^1^ with corresponding GWAS IDs. Another 91 inflammatory cytokine GWAS were derived from a genome-wide protein quantitative trait locus study of circulating inflammatory proteins in 14,824 individuals of European ancestry (Zhao et al., 2023), including 11 study cohorts such as The INTERVAL study (median age 61 years). GWAS data for these 91 inflammatory factors are available through the GWAS Catalog project^2^ with corresponding GWAS IDs. The GWAS data for iNPH was obtained from the European cohort: the FinnGen study, which serves as a large-scale biobank resource with specific characteristics of the Nordic healthcare system and population structure, facilitating a broad spectrum of genetic discoveries. The latest R10 release included a total of 1,456 cases and 409,726 control samples (Kurki et al., 2023). iNPH diagnosis was determined according to the International Classification of Diseases-10th Revision standard, code G91.2.

### 2.3 Selection of IVs

When using circulating inflammatory biomarkers as exposure factors, the selected single nucleotide polymorphisms (SNPs) were required to meet the criterion of genome-wide significance association with each factor being <5 × 10^−6^, to ensure that the inflammatory biomarkers had sufficiently strongly associated SNPs with iNPH (Yeung and Schooling, 2021). When iNPH was used as an exposure factor, we adjusted the significance level to 5 × 10^−8^ (Deng et al., 2023). Subsequently, we clumped the selected SNPs with an *r*^2^ = value of 0.001 and a distance of 10,000 kbto minimize the impact of linkage disequilibrium on the results (Chen et al., 2023). Additionally, a minor allele frequency (MAF) > 0.01 (mutations present in >1% of the population) was required (Xiao et al., 2023). The variation explained by each genetic instrument was determined using the following formula: 2 × EAF × (1 − EAF) × beta^2^, where EAF is the effect allele frequency and beta is the estimated effect. The total variance explained (*R*^2^) for each exposure was computed as the cumulative value for each SNP (Papadimitriou et al., 2020). After extracting SNPs for each exposure factor from the results, they were removed if IVs were not found in the result data or if they had palindromic sequences. *F*-statistics ($F=\left(\frac{{\mathrm{beta}}^{2}}{{se}^{2}}\right)$, where beta is the estimated effect and se is the standard error) were used to assess the strength of individual IV, and instruments with *F* < 10 were considered weak and excluded (Burgess et al., 2011).

### 2.4 Statistical analysis

Causal associations were primarily investigated using the inverse variance-weighted (IVW) method, supplemented by MR-Egger, weighted median, simple mode, and weighted mode analyses (Fang et al., 2023). Heterogeneity in the results was assessed using the Cochran *Q* test (Xiang et al., 2022). Horizontal pleiotropy was evaluated through the MR-Egger intercept test and the MR pleiotropy residual sum and outliers (MR-PRESSO) test (Li et al., 2021). Sensitivity was analyzed via leave-one-out analysis (Li et al., 2023). After Bonferroni correction for multiple comparisons, statistical significance was defined as *p* < 3.8 × 10^−4^ (0.05/132), respectively. *p*-values ranging from 3.8 × 10^−4^ to 0.05 were considered suggestive associations (Fang et al., 2024). For exposures with nominal significance (*p* < 0.05) in the MR analysis, systematic screening for potential phenotypes correlated with confounders was conducted through LDlink.^3^ For binary exposure factor variables, we used the exposure factor’s odds to estimate its causal effect on the outcome (Burgess and Labrecque, 2018). Furthermore, we conducted a meta-analysis of all results for the same inflammatory biomarkers from both data sources (Zhong et al., 2023). Reverse MR analyses were performed to minimize bias from reverse causality. All statistical analyses were performed using R-4.3.2 with R packages, including the TwoSampleMR, MendelianRandomization, and MR-PRESSO packages.

## 3 Results

In total, 133 GWASs (132 GWASs of inflammatory biomarkers and one GWAS of iNPH) were enrolled in this MR study. Supplementary Table 1 provides the summary information of the enrolled GWAS studies. After excluding unmatched SNPs, variation explained by individual exposures ranged from 0.13% for CCL28 to 11.8% for VEGF. *F*-statistics were 20.8–1472.9, indicating the robustness of all SNPs. For further details, refer to Supplementary Table 2.

### 3.1 Influence of inflammatory biomarkers on iNPH

**Figure 2:**
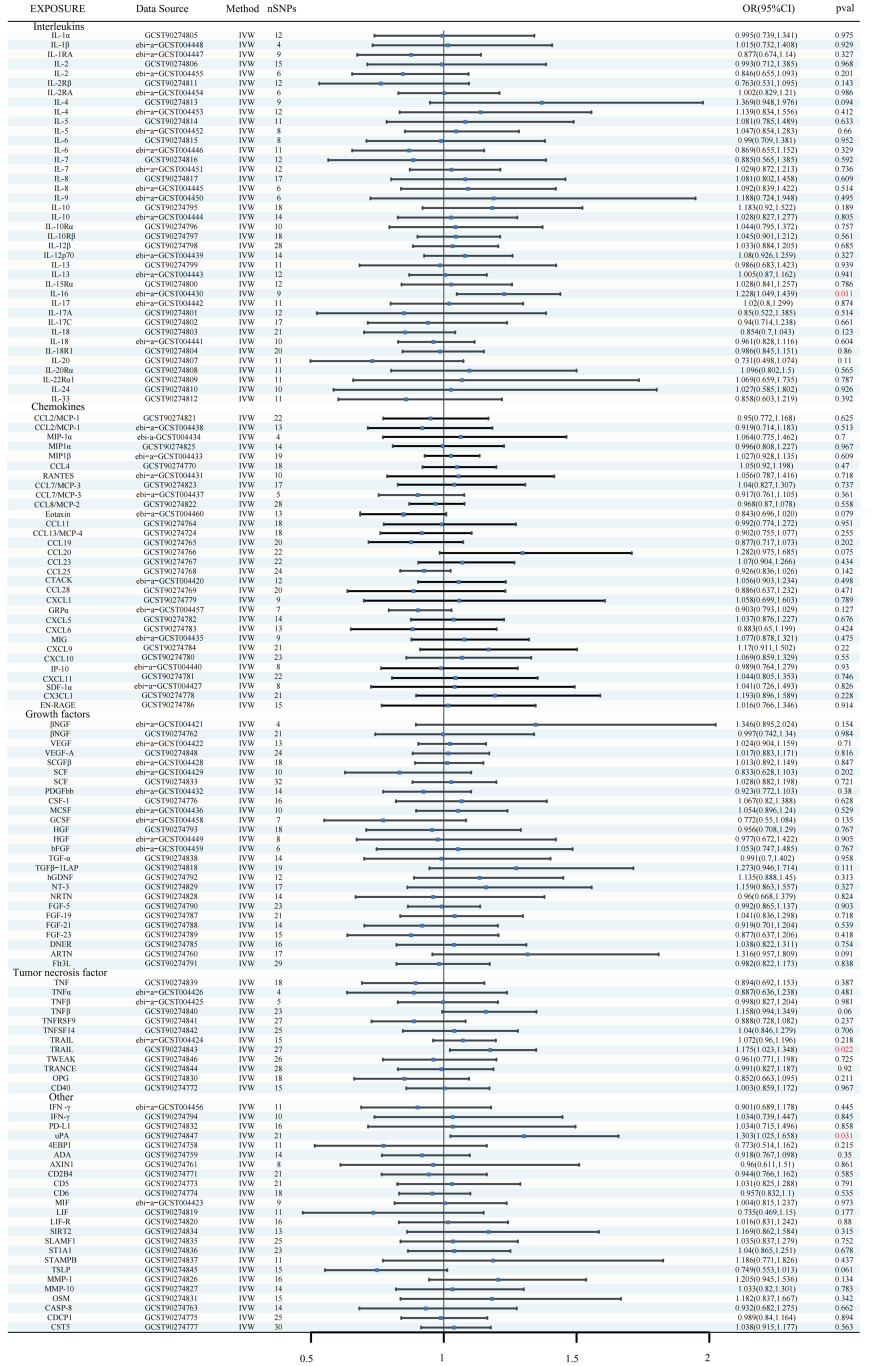
Forest plots of causal relationships between 132 inflammatory biomarkers and iNPH based on IVW analysis results from forward MR analysis. SNP, Single-nucleotide polymorphism; IVW, Inverse variance weighted; OR, Odds Ratio; CTACK, Cutaneous T-cell attracting; VEGF, Vascular endothelial growth factor; MIF, Macrophage Migration Inhibitory Factor; TRAIL, TNF-related apoptosis inducing ligand; TNF, Tumor necrosis factor; TGF, Transforming growth factor; TNFRSF, Tumor necrosis factor receptor superfamily member; TRANCE, TNF-related activation cytokine; SDF, Stromal-cell-derived factor; SCGF, Stem cell growth factor; SCF, Stem cell factor; CSF, Macrophage colony-stimulating factor; HGF, Hepatocyte growth factor; NGF, nerve growth factor; FGF, Fibroblast growth factor; LIF, Leukemia inhibitory factor; SCF, Stem cell factor; IL, Interleukin; IL-1RA, Interleukin-1-receptor antagonist; IL-10R, Interleukin-10 receptor; RANTES, Regulated on activation, normal T cell expressed and secreted; PDGFbb, Platelet-derived growth factor BB; MIP, Macrophage inflammatory protein; MIG, Monokine induced by gamma interferon; MCSF, Macrophage colony stimulating factor; MCP, Monocyte chemoattractant protein; CCL, C-C motif chemokine; IP-10, Interferon gamma-induced protein 10; IFN, Interferon; GRP, Growth-regulated protein; GCSF, Granulocyte-colony stimulating factor; Eotaxin, Eotaxin; CCL11, Eotaxin-1; 4EBP1, Eukaryotic translation initiation factor 4E-binding protein 1; ADA, Adenosine Deaminase; ARTN, Artemin; AXIN1, Axin-1; CASP-8, Caspase 8; CD2B4, Natural killer cell receptor 2B4; CD40, CD40L receptor; CD5, T-cell surface glycoprotein CD5; CD6, T-cell surface glycoprotein CD6 isoform; CDCP1, CUB domain-containing protein 1; CST5, Cystatin D; CX3CL1, Fractalkine; CXCL, C-X-C motif chemokine; DNER, Delta and Notch-like epidermal growth factor related receptor; EN-RAGE, Protein S100-A12; FIt3L, Fms-related tyrosine kinase 3 ligand; hGDNF, human Glial cell line-derived neurotrophic factor; TGFβ1-LAP, Latency-associated peptide transforming growth factor beta 1; MMP-1, Matrix metalloproteinase-1; NRTN, Neurturin; NT-3, Neurotrophin-3; OPG, Osteoprotegerin; OSM, Oncostatin-M; PD-L1, Programmed cell death 1 ligand 1; SIRT2, SIR2-like protein 2; SLAMF1, Signaling lymphocytic activation molecule; SLAMF1, Signaling lymphocytic activation molecule; TSLP, Thymic stromal lymphopoietin; TRAIL, TNF-related apoptosis ligand; TWEAK, Tumor necrosis factor (Ligand) superfamily member 12; uPA, Urokinase-type plasminogen activator.

**Figure 3:**
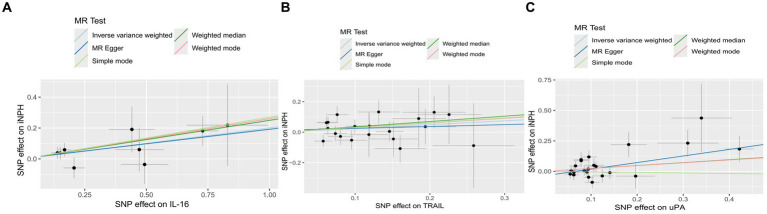
The scatter plots of Mendelian randomization analyses for IL-16 **(A)**, TRAIL **(B)** and uPA **(C)** in iNPH. MR, Mendelian randomization, SNP, Single-nucleotide polymorphism; iNPH, idiopathic Normal pressure hydrocephalus; IL-16, Interleukin-16; TRAIL, TNF-related apoptosis ligand; uPA, Urokinase-type plasminogen activator.

**Figure 4:**
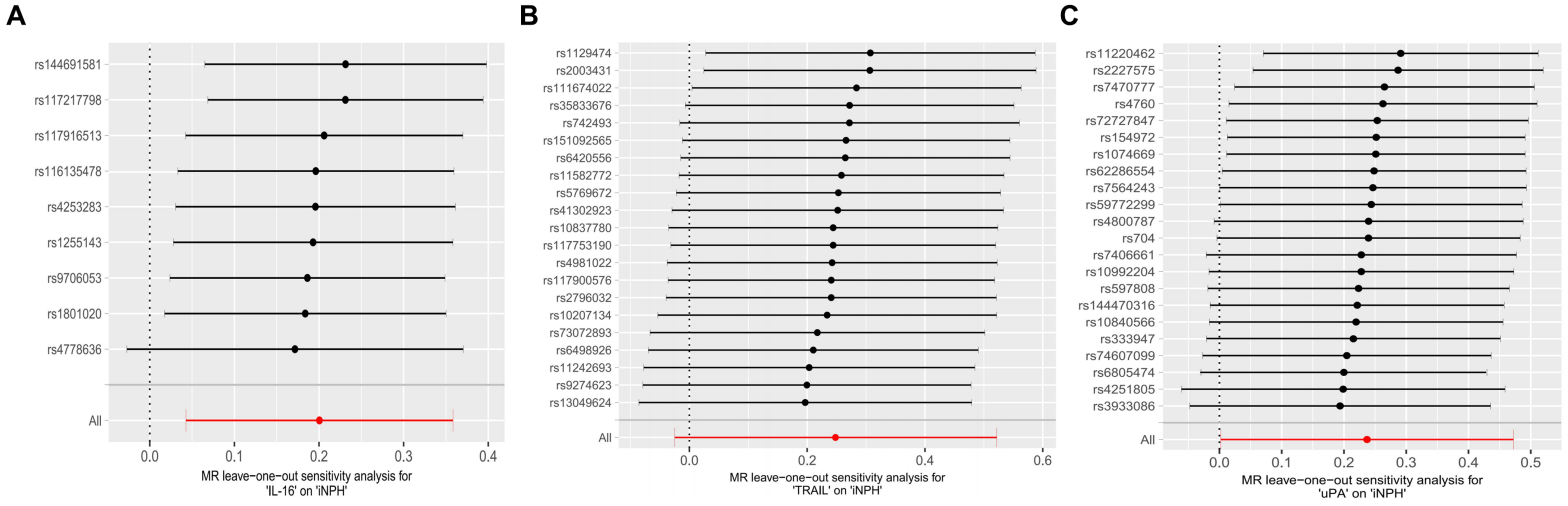
Forest plots of causal relationships between three inflammatory biomarkers —, namely IL-16 **(A)**, TRAIL **(B)**, and uPA **(C)** and iNPH based on ‘leave-one-out’ analysis results from forward MR analysis. MR, Mendelian randomization, SNP, Single-nucleotide polymorphism; iNPH, idiopathic Normal pressure hydrocephalus; IL-16, Interleukin-16; TRAIL, TNF-related apoptosis ligand; uPA, Urokinase-type plasminogen activator.

**Figure 5:**
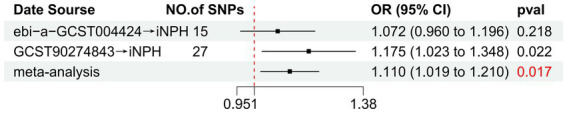
Forest plot of the relationship between TRAIL and iNPH based on meta-analysis results from the forward MR analysis. MR, Mendelian randomization, SNP, Single-nucleotide polymorphism; OR, Odds Ratio; iNPH, idiopathic Normal pressure hydrocephalus; TRAIL; TNF-related apoptosis ligand.

Using the IVW method as the primary MR analysis, no significant casual effect was observed after Bonferroni adjustment. However, a suggestive association between IL-16 and iNPH, with OR of 1.228 [95% CI, 1.049–1.439, *p* = 0.011] was identified in IVW analysis. TRAIL and uPA were found to be suggestively positively associated with iNPH, with ORs of 1.175 [95% CI = 1.023–1.348, *p* = 0.022] and 1.303 [95% CI = 1.025–1.658, *p* = 0.031], respectively. Detailed results of the IVW, MR-Egger, weighted median, simple mode and weighted mode analyses for the 132 inflammatory biomarkers in Figure 2 and Supplementary Table 3. The scatter plots of Mendelian randomization analyses for IL-16, TRAIL and uPA in iNPH are exhibited in Figure 3. Cochran’s *Q* test did not detect evidence of heterogeneity (*Q* value = 4.675, *p* = 0.792; *Q* value = 19.901, *p* = 0.702; *Q* value = 30.205, *p* = 0.088). MR-Egger intercept analysis detected no potential horizontal pleiotropy (intercept = 0.003, *p* = 0.933; intercept = 0.032, *p* = 0.142; intercept = −0.036, *p* = 0.197). Similarly, MR-PRESSO results indicated no horizontal multidirectionality in the MR analysis (SSobs = 5.615, *p* = 0.829; SSobs = 23.055, *p* = 0.644; SSobs = 33.108, *p* = 0.094). A summary of heterogeneity, pleiotropy, and MR-PRESSO test results is presented in Table 1. Additionally, the “leave-one-out” analysis demonstrated the robustness of the MR analysis (Figure 4). Furthermore, to enhance result robustness, a meta-analysis of the same inflammatory biomarkers from both data sources was performed. Meta-analysis showed that elevated TRAIL [OR: 1.111, 95% CI: 1.019–1.210, *p* = 0.017] levels were associated with increased iNPH risk based on IVW methods after combining two MR results from different data sources (IEU database and GWAS catalog database). The causality of other inflammatory factors with iNPH was not found at the meta-analysis stage (Figure 5 and Supplementary Table 4).

**Table 1:** Heterogeneity and horizontal pleiotropy test results for three inflammatory biomarkers and iNPH from forward MR analysis.

Exposure	Outcome	Heterogeneity test				Pleiotropy test			MR-PRESSO	
		Method	*Q*	Q_df	Q_pval	Egger intercept	se	pval	RSSobs	Global Test$Pvalue
IL-16	iNPH	MR Egger	4.67	7	0.700	0.003	0.04	0.93	5.615	0.829
		IVW	4.68	8	0.792					
TRAIL	iNPH	MR Egger	17.59	23	0.779	0.032	0.02	0.14	23.055	0.644
		IVW	19.90	24	0.702					
uPA	iNPH	MR Egger	27.74	20	0.116	−0.036	0.03	0.2	33.108	0.094
		IVW	30.20	21	0.088					

MR, Mendelian randomization, IVW, Inverse variance weighted; iNPH, idiopathic Normal pressure hydrocephalus; IL-16, Interleukin-16; TRAIL, TNF-related apoptosis ligand; uPA, Urokinase-type plasminogen activator.

### 3.2 Influence of iNPH on inflammatory biomarkers

**Figure 6:**
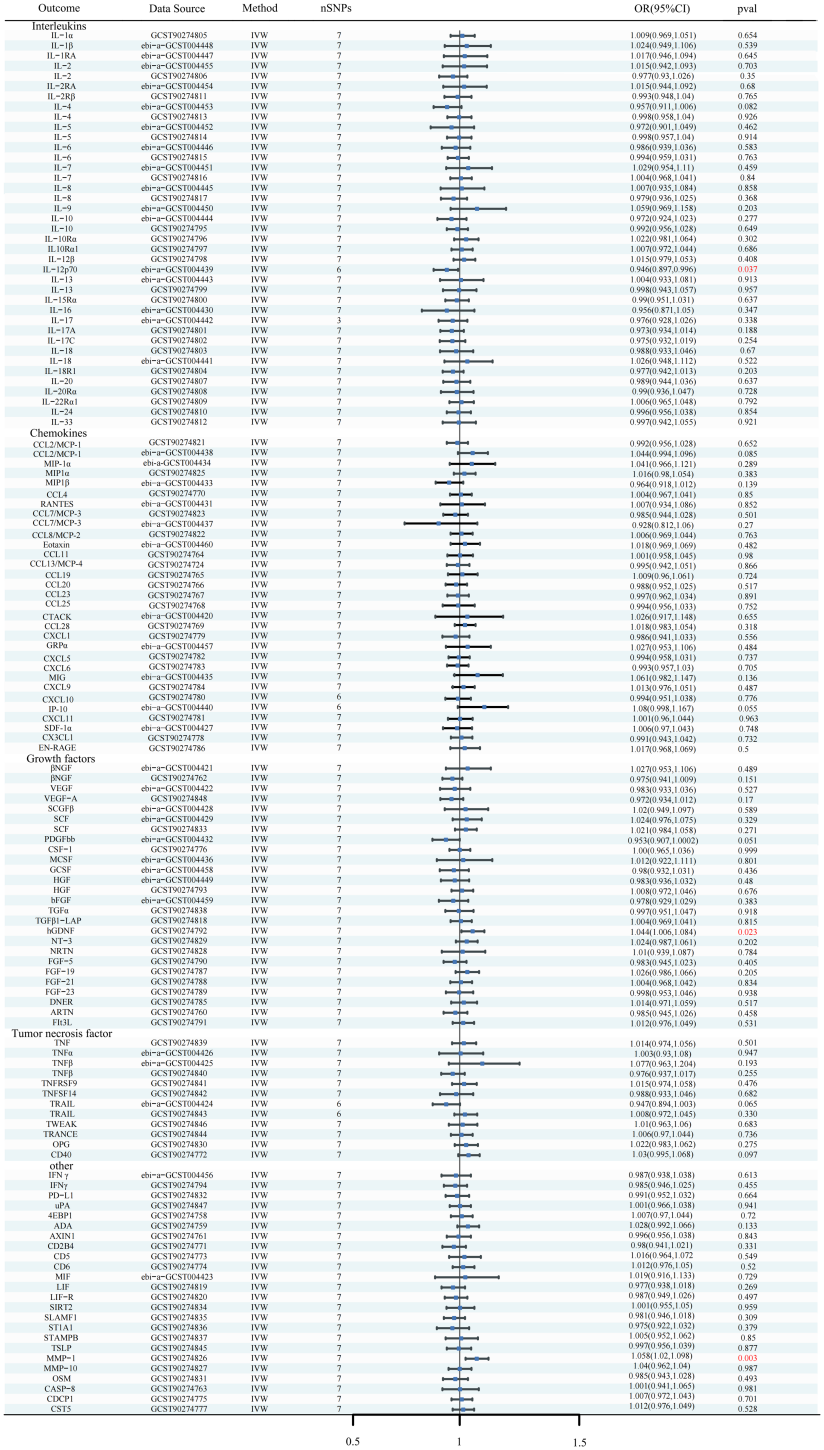
Forest plots of the causal relationships between iNPH and 132 inflammatory biomarkers based on IVW analysis results from reverse MR analysis. SNP, Single-nucleotide polymorphism; IVW, Inverse variance weighted; OR, Odds Ratio; CTACK, Cutaneous T-cell attracting; VEGF, Vascular endothelial growth factor; MIF, Macrophage Migration Inhibitory Factor; TRAIL, TNF-related apoptosis inducing ligand; TNF, Tumor necrosis factor; TGF, Transforming growth factor; TNFRSF, Tumor necrosis factor receptor superfamily member; TRANCE, TNF-related activation cytokine; SDF, Stromal-cell-derived factor; SCGF, Stem cell growth factor; SCF, Stem cell factor; CSF, Macrophage colony-stimulating factor; HGF, Hepatocyte growth factor; NGF, nerve growth factor; FGF, Fibroblast growth factor; LIF, Leukemia inhibitory factor; SCF, Stem cell factor; IL, Interleukin; IL-1RA, Interleukin-1-receptor antagonist; IL-10R, Interleukin-10 receptor; RANTES, Regulated on activation, normal T cell expressed and secreted; PDGFbb, Platelet-derived growth factor BB; MIP, Macrophage inflammatory protein; MIG, Monokine induced by gamma interferon; MCSF, Macrophage colony stimulating factor; MCP, Monocyte chemoattractant protein; CCL, C-C motif chemokine; IP-10, Interferon gamma-induced protein 10; IFN, Interferon; GRP, Growth-regulated protein; GCSF, Granulocyte-colony stimulating factor; Eotaxin, Eotaxin; CCL11, Eotaxin-1; 4EBP1, Eukaryotic translation initiation factor 4E-binding protein 1; ADA, Adenosine Deaminase; ARTN, Artemin; AXIN1, Axin-1; CASP-8, Caspase 8; CD2B4, Natural killer cell receptor 2B4; CD40, CD40L receptor; CD5, T-cell surface glycoprotein CD5; CD6, T-cell surface glycoprotein CD6 isoform; CDCP1, CUB domain-containing protein 1; CST5, Cystatin D; CX3CL1, Fractalkine; CXCL, C-X-C motif chemokine; DNER, Delta and Notch-like epidermal growth factor related receptor; EN-RAGE, Protein S100-A12; FIt3L, Fms-related tyrosine kinase 3 ligand; hGDNF, human Glial cell line-derived neurotrophic factor; TGFβ1-LAP, Latency-associated peptide transforming growth factor beta 1; MMP-1, Matrix metalloproteinase-1; NRTN, Neurturin; NT-3, Neurotrophin-3; OPG, Osteoprotegerin; OSM, Oncostatin-M; PD-L1, Programmed cell death 1 ligand 1; SIRT2, SIR2-like protein 2; SLAMF1, Signaling lymphocytic activation molecule; SLAMF1, Signaling lymphocytic activation molecule; TSLP, Thymic stromal lymphopoietin; TWEAK, Tumor necrosis factor (Ligand) superfamily member 12; uPA, Urokinase-type plasminogen activator.

**Figure 7:**
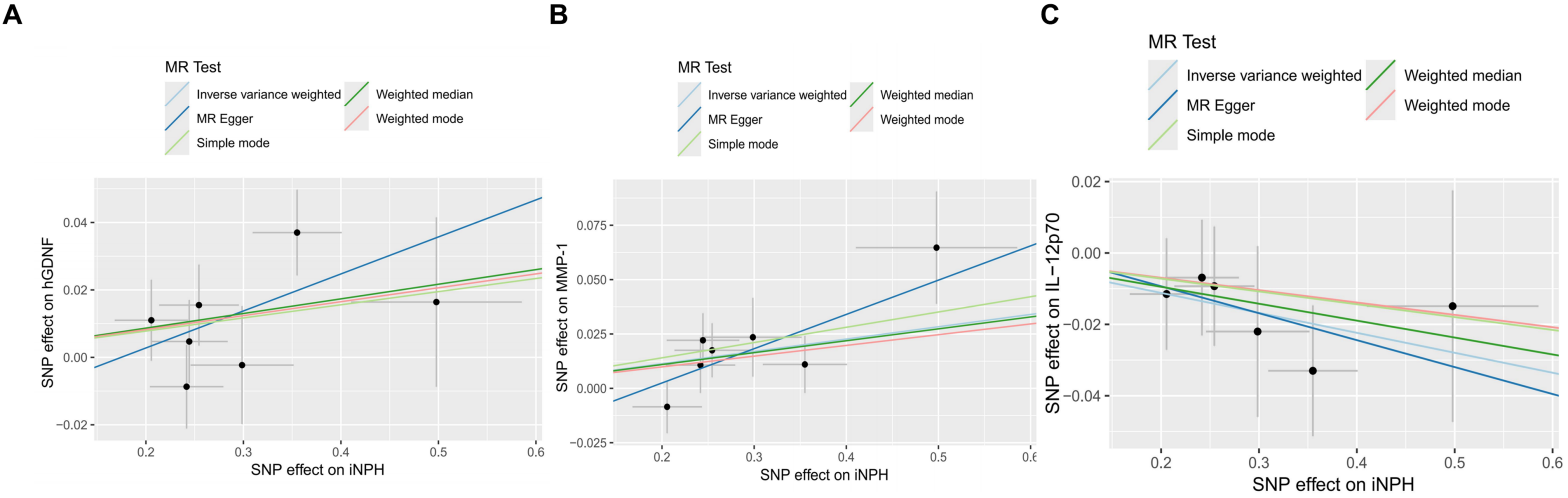
The Scatter plots of Mendelian randomization analyses between iNPH and inflammatory biomarkers —, namely hGDNF **(A)**, MMP-1 **(B)** and IL-12p70 **(C)**. MR, Mendelian randomization, SNP, Single-nucleotide polymorphism; iNPH, idiopathic Normal pressure hydrocephalus; hGDNF, human Glial cell line-derived neurotrophic factor; MMP-1, Matrix metalloproteinase-1; IL-12p70, Interleukin-12p70.

**Figure 8:**
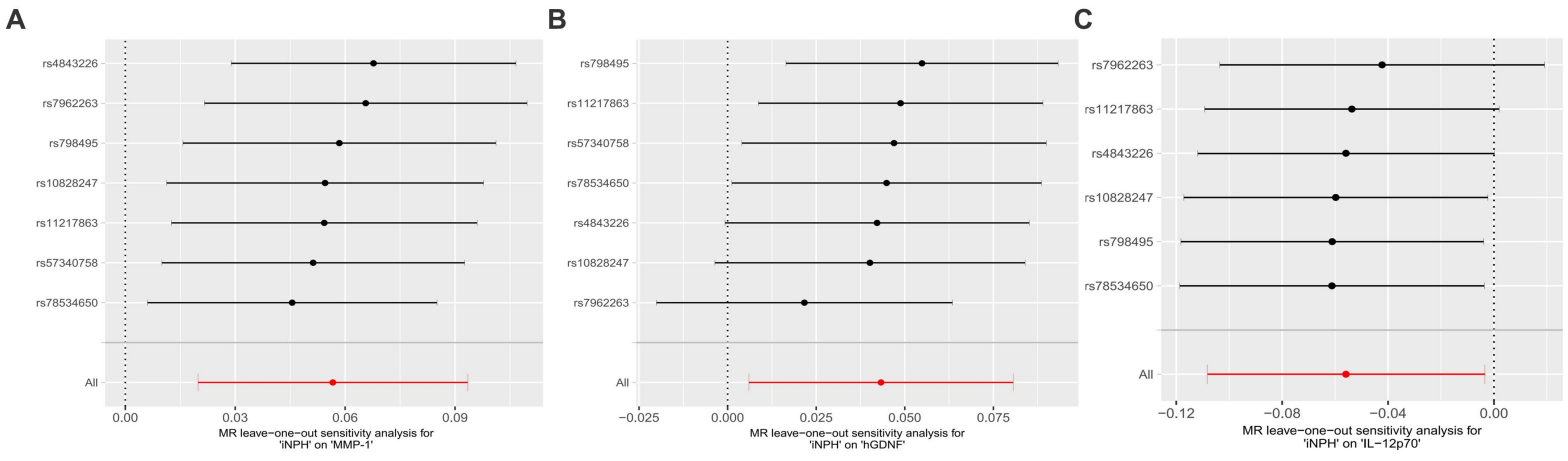
Forest plots of causal relationships between iNPH and three inflammatory biomarkers —, namely MMP-1 **(A)**, hGDNF **(B)** and IL-12p70 **(C)** based on ‘leave-one-out’ analysis results from reverse MR analysis. MR, Mendelian randomization, SNP, Single-nucleotide polymorphism; iNPH, idiopathic Normal pressure hydrocephalus; hGDNF, Glial cell line-derived neurotrophic factor; MMP-1, Matrix metalloproteinase-1; IL-12p70, Interleukin-12p70.

In reverse MR analysis, a suggestive association between genetically predicted iNPH and inflammatory biomarkers was found. The IVW analysis revealed a suggestive positive correlation between iNPH and MMP-1, with an OR of 1.058 [95% CI = 1.020–1.098, *p* = 0.003]. A similar association was observed for hGDNF, with an OR of 1.044 [95% CI = 1.006–1.084, *p* = 0.022], whereas IL-12p70 exhibited a nominal negative correlation with iNPH, with OR of 0.897 [95% CI = 0.946–0.997, *p* = 0.037]. Figure 6 and Supplementary Table 5 provide the results of IVW, MR-Egger, weighted median, simple mode and weighted mode analyses. Scatter plots of Mendelian randomization (MR) analyses between iNPH and inflammatory biomarkers are exhibited in Figure 7. No evidence of pleiotropy and heterogeneity was observed in these results. Additionally, MR-PRESSO test results showed no horizontal multidirectionality in the MR analysis (SSobs = 1.393, *p* = 0.984; SSobs = 9.982, *p* = 0.354; SSobs = 7.884, *p* = 0.4814; SSobs = 8.113, *p* = 0.453). Table 2 provides a summary of the heterogeneity, pleiotropy, and MR-PRESSO test results. Furthermore, the “leave-one-out” analysis demonstrated the robustness of the MR analysis (Figure 8). Again, after meta-analysis of all results for the same inflammatory biomarkers from both data sources, no positive result was observed (Supplementary Table 6).

**Table 2:** Heterogeneity and horizontal pleiotropy test result for iNPH and three inflammatory biomarkers in the reverse MR analysis.

Exposure	Outcome	Heterogeneity test				Pleiotropy test			MR-PRESSO	
		Method	*Q*	Q_df	Q_pval	Egger intercept	se	pval	RSSobs	Global Test$Pvalue
iNPH	hGDNF	MR Egger	5.69	5	0.337	−0.019	0.02	0.44	9.982	0.354
		IVW	6.49	6	0.371					
iNPH	MMP-1	MR Egger	4.20	5	0.521	−0.029	0.02	0.24	7.884	0.481
		IVW	5.95	6	0.429					
iNPH	IL-12p70	MR Egger	0.94	4	0.901	0.006	0.030	0.86	1.622	0.955
		IVW	0.95	5	0.964					

MR, Mendelian randomization, IVW, Inverse variance weighted; iNPH, idiopathic Normal pressure hydrocephalus; hGDNF, human Glial cell line-derived neurotrophic factor; MMP-1, Matrix metalloproteinase-1; IL-12p70, Interleukin-12p70.

## 4 Discussion

We used two-sample bidirectional MR combined with meta-analysis as a novel approach to examine the causal relationship between inflammatory biomarkers and iNPH risk from a genetic perspective. Notably, we included novel factors, such as TRAIL and uPA, which had not been previously researches, yielding results not previously reported. These findings offer potential insights into how inflammation contributes to the onset and progression of iNPH. Although no casual effects were observed after Bonferroni’s adjustment in the MR analysis, genetically predicted IL-16, uPA, and TRAIL levels appeared to be positively associated with iNPH. Moreover, we observed that iNPH onset may correlate with elevated MMP-1 and hGDNF levels, alongside reduced IL-12p70 levels. These outcomes remained robust following sensitivity analyses. Therefore, these findings provide valuable insights into iNPH prevention and treatment.

Circulating proteins play pivotal roles in inflammation and a broad range of diseases, and systemic inflammation markedly impacts brain function (Lucin and Wyss-Coray, 2009; Wyss-Coray, 2016). Since its proposal in 1965, iNPH has garnered attention due to its reversible dementia features ameliorated by shunt surgery (Adams et al., 1965). In recent years, researches have highlighted substantial reductions in white matter volume and increases in the volume and ratio of CSF as well as the total intracranial volume, with neurodegenerative CSF biomarkers showing correlations with preoperative and postoperative cognition, offering insights into neuropathological processes (Lukkarinen et al., 2022). However, accurate iNPH diagnosis remains crucial, given its clinical and laboratory similarities to other brain degenerative diseases, including Alzheimer’s disease (Li et al., 2023).

IL-16, a chemically induced immunomodulatory cytokine observed across autoimmune and inflammatory diseases, triggers cellular responses by interacting with membrane-expressed CD4 (Center et al., 2001), thereby elevating the levels of other inflammatory cytokines, such as IL-1β, IL-6, and TNFα, via monocytes (Mathy et al., 2000), consistent with previous findings regarding CSF biomarkers. Elevated IL-16 levels have been linked to increased risk of white matter lesions in patients with mild cognitive impairment (Kouchaki et al., 2022), and we speculate that they may be associated with decrease white matter and heightened dementia risk in patients with iNPH (Gertje et al., 2023). TRAIL, a member of the TNF superfamily released by microglia, is implicated in the pathophysiology of multiple sclerosis, bacterial meningitis, HIV encephalitis, stroke, and AD (Dörr et al., 2002). Moreover, TRAIL promotes apoptosis of parenchymal cells in disease by interacting with TRAIL death receptors expressed on the cells (Hoffmann et al., 2009). Our findings suggest that elevated TRAIL levels may serve as a risk factor for iNPH, although the precise pathogenesis remains unclear. Blood–brain barrier impairment is a feature of various neurodegenerative diseases (Pisani et al., 2012; Janelidze et al., 2017). uPA, as a serine protease, induces astrocyte activation by activating plasminogen in the central nervous system (Diaz et al., 2021). Its role in iNPH has not been elucidated, but its increased expression after peripheral thermal injury suggests BBB involvement (Kataoka et al., 2000; Patel et al., 2008). Understanding the mechanisms by which these biomarkers function in the context of disease remain warrants further investigation.

MMPs may play a pathogenic role in neurological disorders (Romi et al., 2012). Metalloproteinase activity regulates critical immunity signal transduction pathways, such as TNF and IL-6 receptors, thereby controlling the dynamics, amplitude, and combinations of molecular signals in tissues (Khokha et al., 2013). Previous studies have revealed that MMP-1 levels in lumbar CSF after shunt surgery are higher than those before surgery in patients with iNPH (Minta et al., 2021). Further research is warranted to elucidate the precise mechanism underlying MMP-1 changes and their impact on iNPH development. Notably, the relationship between hGDNF and iNPH remains unexplored. As a member of the transforming growth factor-β superfamily, hGDNF promotes the survival and morphological differentiation of dopaminergic neurons (Lin et al., 1993). Our MR results suggest that iNPH is associated with high hGDNF levels, possibly reflecting a form of negative feedback regulation in the body’s self-protection mechanism. However, this hypothesis requires testing in future researches. IL-12p70, a myeloid cell-secreted cytokine, has been implicated as a marker of resilience against downstream pathological events (Yang et al., 2022). Higher IL-12p70 levels were associated with slower cognitive decline at higher amyloid-*β* levels (Leinonen et al., 2011), and high IL-12p70 was associated with reduced tau protein and neurodegeneration in participants with high amyloid-*β* (Said et al., 2022), a conclusion highlighting the possible association between iNPH and neurodegenerative disease. In addition, immunogold cytochemistry analysis of AQP4 by cortical brain biopsies showed that AQP4 density decreased in the perivascular astroglial endfeet of iNPH brains. This association may indicate a role of inflammation-induced AQP4 depolarization in the pathogenesis of iNPH. Reducing perivascular AQP4 expression reduces glymphatic fluid flux, thereby exacerbating amyloid-*β* accumulation and ventricle enlargement (Reeves et al., 2020).

Several limitations of this study must be acknowledged. First, given the higher statistical power of the IVW method compared with the other four MR Methods, it was chosen as the primary MR analysis method. We ensured consistency in beta direction across different methods, enhancing the references value our results. Second, despite using the latest and largest iNPH GWAS dataset from FinnGen, potential biases persist. Given the lack of GWAS datasets currently available for iNPH and the potentially differing distribution of genetic polymorphisms across populations, questions may be raised regarding the consistency of results of studies with aggregated GWAS statistics limited to European ancestry across populations. We found causal inconsistencies between inflammatory biomarkers and iNPH across different databases. For example, a causal relationship between TRAIL and iNPH was found in the GWAS catalog database, but not in the IEU database. We hypothesize that this is due to differences in the composition of the gene pool, since Finland is a well-established genetic isolate and its unique gene pool distinguishes Finns from other Europeans, which may lead to causal inconsistencies between the IEU database and the GWAS catalog database. Third, it is important to note that the interaction between iNPH and inflammatory factors found in this study may only represent the preclinical iNPH phase, and because the GWAS population with inflammatory factors is relatively young and most participants have not yet reached the usual age for iNPH diagnosis, the effect between the two may not be accurately reflected. Moreover, the possibility of secondary normal-pressure hydrocephalus among iNPH cases may have influenced our findings. Previous researches have employed more lenient thresholds (*p* < 5 × 10^−6^) for studying the relationship between inflammatory biomarkers and disease because more stringent thresholds (*p* < 5 × 10^−8^) result in fewer available IVs. As a more lenient threshold was used in the present study, this may have introduced false positive SNPs with low statistical power and insufficient sensitivity analysis. Notably, rigorous sensitivity analysis detected no significant heterogeneity or horizontal pleiotropy. We only tested the causal effect of systemic inflammation on the risk of iNPH, but not their effect on disease progression, as there is currently no way to do so. Ultimately, the absence of a causal relationship between iNPH and TNFα, IL-6, and IL-1β, could be attributed to the relatively small number of SNPs included in MR studies, and suggesting the need for larger sample sizes in further investigations.

## 5 Conclusion

This study yielded suggestive evidence warranting further exploration in iNPH research. Although our findings offer new avenues for investigation, confirmation and integration into clinical diagnostic procedures and treatment protocols necessitate additional research.

## Data availability statement

The original contributions presented in the study are included in the article/Supplementary material, further inquiries can be directed to the corresponding authors.

## Ethics statement

Ethical approval was not required for the study involving humans in accordance with the local legislation and institutional requirements. Written informed consent to participate in this study was not required from the participants or the participants’ legal guardians/next of kin in accordance with the national legislation and the institutional requirements.

## Author contributions

JL: Conceptualization, Data curation, Formal analysis, Investigation, Methodology, Software, Validation, Writing – original draft, Writing – review & editing. XW: Conceptualization, Data curation, Formal analysis, Investigation, Methodology, Software, Validation, Writing – original draft, Writing – review & editing. FX: Conceptualization, Data curation, Formal analysis, Investigation, Methodology, Software, Validation, Writing – original draft, Writing – review & editing. CR: Data curation, Methodology, Writing – review & editing. YG: Formal analysis, Project administration, Writing – review & editing. ZS: Resources, Supervision, Writing – review & editing. SC: Formal analysis, Funding acquisition, Project administration, Writing – review & editing. QL: Formal analysis, Funding acquisition, Project administration, Writing – review & editing, Resources, Supervision, Visualization.

## Footnotes

^1^ https://gwas.mrcieu.ac.uk/

^2^ https://www.ebi.ac.uk/gwas/

^3^ https://ldlink.nci.nih.gov/

## References

[ref1] Adams R. D. Fisher C. M. Hakim S. Ojemann R. G. Sweet W. H. (1965). Symptomatic occult hydrocephalus with “normal” cerebrospinal-fluid pressure. A treatable syndrome. N. Engl. J. Med. 273, 117–126. doi: 10.1056/NEJM196507152730301, PMID: 14303656

[ref2] Ahola-Olli A. V. Würtz P. Havulinna A. S. Aalto K. Pitkänen N. Lehtimäki T.. (2017). Genome-wide association study identifies 27 loci influencing concentrations of circulating cytokines and growth factors. Am. J. Hum. Genet. 100, 40–50. doi: 10.1016/j.ajhg.2016.11.007, PMID: 27989323 PMC5223028

[ref3] Bonney P. A. Briggs R. G. Wu K. Choi W. Khahera A. Ojogho B.. (2022). Pathophysiological mechanisms underlying idiopathic normal pressure hydrocephalus: a review of recent insights. Front. Aging Neurosci. 14:866313. doi: 10.3389/fnagi.2022.866313, PMID: 35572128 PMC9096647

[ref4] Bowden J. Holmes M. V. (2019). Meta-analysis and Mendelian randomization: a review. Res. Synth. Methods 10, 486–496. doi: 10.1002/jrsm.1346, PMID: 30861319 PMC6973275

[ref5] Braun M. Boström G. Ingelsson M. Kilander L. Löwenmark M. Nyholm D.. (2023). Levels of inflammatory cytokines MCP-1, CCL4, and PD-L1 in CSF differentiate idiopathic normal pressure hydrocephalus from neurodegenerative diseases. Fluids Barriers CNS 20:72. doi: 10.1186/s12987-023-00472-x 37833765 PMC10571396

[ref6] Burgess S. Labrecque J. A. (2018). Mendelian randomization with a binary exposure variable: interpretation and presentation of causal estimates. Eur. J. Epidemiol. 33, 947–952. doi: 10.1007/s10654-018-0424-6, PMID: 30039250 PMC6153517

[ref7] Burgess S. Thompson S. G. CRP CHD Genetics Collaboration (2011). Avoiding bias from weak instruments in Mendelian randomization studies. Int. J. Epidemiol. 40, 755–764. doi: 10.1093/ije/dyr036, PMID: 21414999

[ref8] Center D. M. Cruikshank W. W. Parada N. A. Ryan T. Theodore A. C. Viglianti G.. (2001). Measurement of interleukin 16. Curr. Protoc. Immunol. Chapter 6, 6.23.1–6.23.14. doi: 10.1002/0471142735.im0623s22 18432814

[ref9] Chen Y. Shen J. Wu Y. Ni M. Deng Y. Sun X.. (2023). Tea consumption and risk of lower respiratory tract infections: a two-sample Mendelian randomization study. Eur. J. Nutr. 62, 385–393. doi: 10.1007/s00394-022-02994-w, PMID: 36042048 PMC9427168

[ref10] Czubowicz K. Głowacki M. Fersten E. Kozłowska E. Strosznajder R. P. Czernicki Z. (2017). Levels of selected pro- and anti-inflammatory cytokines in cerebrospinal fluid in patients with hydrocephalus. Folia Neuropathol. 55, 301–307. doi: 10.5114/fn.2017.72389, PMID: 29363904

[ref11] Deng Z. Wang H. Huang K. Li Y. Ran Y. Chen Y.. (2023). Association between vascular risk factors and idiopathic normal pressure hydrocephalus: a Mendelian randomization study. J. Neurol. 270, 2724–2733. doi: 10.1007/s00415-023-11604-6, PMID: 36773060

[ref12] Diaz A. Martin-Jimenez C. Xu Y. Merino P. Woo Y. Torre E.. (2021). Urokinase-type plasminogen activator-mediated crosstalk between N-cadherin and β-catenin promotes wound healing. J. Cell Sci. 134:jcs255919. doi: 10.1242/jcs.255919 34085693 PMC8214757

[ref13] Dörr J. Bechmann I. Waiczies S. Aktas O. Walczak H. Krammer P. H.. (2002). Lack of tumor necrosis factor-related apoptosis-inducing ligand but presence of its receptors in the human brain. J. Neurosci. 22:RC209. doi: 10.1523/JNEUROSCI.22-04-j0001.2002 11844843 PMC6757573

[ref14] Fang J. Cao Y. Ni J. (2024). Circulating inflammatory biomarkers and risk of intracranial aneurysm: a Mendelian randomization study. Eur. J. Med. Res. 29:17. doi: 10.1186/s40001-023-01609-2, PMID: 38173028 PMC10763118

[ref15] Fang P. Liu X. Qiu Y. Wang Y. Wang D. Zhao J.. (2023). Exploring causal correlations between inflammatory cytokines and ankylosing spondylitis: a bidirectional Mendelian-randomization study. Front. Immunol. 14:1285106. doi: 10.3389/fimmu.2023.1285106 38054001 PMC10694192

[ref16] Gertje E. C. Janelidze S. van Westen D. Cullen N. Stomrud E. Palmqvist S.. (2023). Associations between CSF markers of inflammation, White matter lesions, and cognitive decline in individuals without dementia. Neurology 100, e1812–e1824. doi: 10.1212/WNL.0000000000207113, PMID: 36882326 PMC10136007

[ref17] Hoffmann O. Zipp F. Weber J. R. (2009). Tumour necrosis factor-related apoptosis-inducing ligand (TRAIL) in central nervous system inflammation. J. Mol. Med. 87, 753–763. doi: 10.1007/s00109-009-0484-x, PMID: 19449143

[ref18] Janelidze S. Hertze J. Nägga K. Nilsson K. Nilsson C. Swedish BioFINDER Study Group. (2017). Increased blood-brain barrier permeability is associated with dementia and diabetes but not amyloid pathology or APOE genotype. Neurobiol. Aging 51, 104–112. doi: 10.1016/j.neurobiolaging.2016.11.017, PMID: 28061383 PMC5754327

[ref19] Kataoka K. Asai T. Taneda M. Ueshima S. Matsuo O. Kuroda R.. (2000). Roles of urokinase type plasminogen activator in a brain stab wound. Brain Res. 887, 187–190. doi: 10.1016/S0006-8993(00)03042-0, PMID: 11134604

[ref20] Khokha R. Murthy A. Weiss A. (2013). Metalloproteinases and their natural inhibitors in inflammation and immunity. Nat. Rev. Immunol. 13, 649–665. doi: 10.1038/nri3499 23969736

[ref21] Kouchaki E. Akbari H. Mahmoudi F. Salehi M. Naimi E. Nikoueinejad H. (2022). Correlation of serum levels of interleukine-16, CCL27, tumor necrosis factor-related apoptosis-inducing ligand, and B-cell activating factor with multiple sclerosis severity. Iran J. Allergy Asthma Immunol. 21, 27–34. doi: 10.18502/ijaai.v21i1.8610, PMID: 35524375

[ref22] Kurki M. I. Karjalainen J. Palta P. Sipilä T. P. Kristiansson K. Donner K. M.. (2023). FinnGen provides genetic insights from a well-phenotyped isolated population. Nature 613, 508–518. doi: 10.1038/s41586-022-05473-8, PMID: 36653562 PMC9849126

[ref23] Leinonen V. Menon L. G. Carroll R. S. Dello Iacono D. Grevet J. Jääskeläinen J. E.. (2011). Cerebrospinal fluid biomarkers in idiopathic normal pressure hydrocephalus. Int. J. Alzheimers Dis. 2011:312526. doi: 10.4061/2011/312526 21660204 PMC3109737

[ref24] Li J. Huang N. Zhang X. Peng J. Huang Q. (2023). Positive association between omega-3/6 polyunsaturated fatty acids and idiopathic normal pressure hydrocephalus: a Mendelian randomization study. Front. Genet. 14:1269494. doi: 10.3389/fgene.2023.1269494, PMID: 38174046 PMC10762850

[ref25] Li H. Liu C. Tai H. Wei Y. Shen T. Yang Q.. (2023). Comparison of cerebrospinal fluid space between probable normal pressure hydrocephalus and Alzheimer’s disease. Front. Aging Neurosci. 15:1241237. doi: 10.3389/fnagi.2023.1241237, PMID: 37693646 PMC10484096

[ref26] Li Y. Lu J. Wang J. Deng P. Meng C. Tang H. (2021). Inflammatory cytokines and risk of ischemic stroke: a Mendelian randomization study. Front. Pharmacol. 12:779899. doi: 10.3389/fphar.2021.779899 35111052 PMC8801801

[ref27] Lin L. F. Doherty D. H. Lile J. D. Bektesh S. Collins F. (1993). GDNF: a glial cell line-derived neurotrophic factor for midbrain dopaminergic neurons. Science 260, 1130–1132. doi: 10.1126/science.8493557 8493557

[ref28] Lolansen S. D. Rostgaard N. Oernbo E. K. Juhler M. Simonsen A. H. MacAulay N. (2021). Inflammatory markers in cerebrospinal fluid from patients with hydrocephalus: a systematic literature review. Dis. Markers 2021, 1–12. doi: 10.1155/2021/8834822 PMC7875647 33613789

[ref29] Lucin K. M. Wyss-Coray T. (2009). Immune activation in brain aging and neurodegeneration: too much or too little? Neuron 64, 110–122. doi: 10.1016/j.neuron.2009.08.039, PMID: 19840553 PMC2834890

[ref30] Lukkarinen H. Jeppsson A. Wikkelsö C. Blennow K. Zetterberg H. Constantinescu R.. (2022). Cerebrospinal fluid biomarkers that reflect clinical symptoms in idiopathic normal pressure hydrocephalus patients. Fluids Barriers CNS 19:11. doi: 10.1186/s12987-022-00309-z, PMID: 35123528 PMC8817565

[ref31] Martín-Láez R. Caballero-Arzapalo H. López-Menéndez L. Á. Arango-Lasprilla J. C. Vázquez-Barquero A. (2015). Epidemiology of idiopathic normal pressure hydrocephalus: a systematic review of the literature. World Neurosurg. 84, 2002–2009. doi: 10.1016/j.wneu.2015.07.005, PMID: 26183137

[ref32] Mathy N. L. Scheuer W. Lanzendörfer M. Honold K. Ambrosius D. Norley S.. (2000). Interleukin-16 stimulates the expression and production of pro-inflammatory cytokines by human monocytes. Immunology 100, 63–69. doi: 10.1046/j.1365-2567.2000.00997.x, PMID: 10809960 PMC2326980

[ref33] Minta K. Jeppsson A. Brinkmalm G. Portelius E. Zetterberg H. Blennow K.. (2021). Lumbar and ventricular CSF concentrations of extracellular matrix proteins before and after shunt surgery in idiopathic normal pressure hydrocephalus. Fluids Barriers CNS 18:23. doi: 10.1186/s12987-021-00256-1 33985551 PMC8120927

[ref34] Mukandala G. Tynan R. Lanigan S. O’Connor J. J. (2016). The effects of hypoxia and inflammation on synaptic signaling in the CNS. Brain Sci. 6:6. doi: 10.3390/brainsci6010006 26901230 PMC4810176

[ref35] Nassar B. R. Lippa C. F. (2016). Idiopathic normal pressure hydrocephalus: a review for general practitioners. Gerontol. Geriatr. Med. 2:2333721416643702. doi: 10.1177/2333721416643702 28138494 PMC5119812

[ref36] Papadimitriou N. Dimou N. Tsilidis K. K. Banbury B. Martin R. M. Lewis S. J.. (2020). Physical activity and risks of breast and colorectal cancer: a Mendelian randomisation analysis. Nat. Commun. 11:597. doi: 10.1038/s41467-020-14389-8, PMID: 32001714 PMC6992637

[ref37] Patel T. H. Sprague S. Lai Q. Jimenez D. F. Barone C. M. Ding Y. (2008). Blood brain barrier (BBB) dysfunction associated with increased expression of tissue and urokinase plasminogen activators following peripheral thermal injury. Neurosci. Lett. 444, 222–226. doi: 10.1016/j.neulet.2008.08.020, PMID: 18718505

[ref38] Pisani V. Stefani A. Pierantozzi M. Natoli S. Stanzione P. Franciotta D.. (2012). Increased blood-cerebrospinal fluid transfer of albumin in advanced Parkinson’s disease. J. Neuroinflammation 9:188. doi: 10.1186/1742-2094-9-188 22870899 PMC3441323

[ref39] Reeves B. C. Karimy J. K. Kundishora A. J. Mestre H. Cerci H. M. Matouk C.. (2020). Glymphatic system impairment in Alzheimer’s disease and idiopathic normal pressure hydrocephalus. Trends Mol. Med. 26, 285–295. doi: 10.1016/j.molmed.2019.11.008, PMID: 31959516 PMC7489754

[ref40] Relkin N. Marmarou A. Klinge P. Bergsneider M. Black P. M. (2005). Diagnosing idiopathic normal-pressure hydrocephalus. Neurosurgery 57, S2-4–S2-16; discussion ii-v. doi: 10.1227/01.NEU.0000168185.29659.C5, PMID: 16160425

[ref41] Romi F. Helgeland G. Gilhus N. E. (2012). Serum levels of matrix metalloproteinases: implications in clinical neurology. Eur. Neurol. 67, 121–128. doi: 10.1159/000334862, PMID: 22262194

[ref42] Said H. M. Kaya D. Yavuz I. Dost F. S. Altun Z. S. Isik A. T. (2022). A comparison of cerebrospinal fluid Beta-amyloid and tau in idiopathic normal pressure hydrocephalus and neurodegenerative dementias. Clin. Interv. Aging 17, 467–477. doi: 10.2147/CIA.S360736, PMID: 35431542 PMC9012339

[ref43] Sanderson E. Glymour M. M. Holmes M. V. Kang H. Morrison J. Munafò M. R.. (2022). Mendelian randomization. Nat. Rev. Methods Primers 2:6. doi: 10.1038/s43586-021-00092-5, PMID: 37325194 PMC7614635

[ref44] Smith G. D. Ebrahim S. (2003). “Mendelian randomization”: can genetic epidemiology contribute to understanding environmental determinants of disease? Int. J. Epidemiol. 32, 1–22. doi: 10.1093/ije/dyg070, PMID: 12689998

[ref45] Tarkowski E. Tullberg M. Fredman P. Wikkelsö C. (2003). Normal pressure hydrocephalus triggers intrathecal production of TNF-alpha. Neurobiol. Aging 24, 707–714. doi: 10.1016/S0197-4580(02)00187-2 12885578

[ref46] Tarnaris A. Kitchen N. D. Watkins L. D. (2009). Noninvasive biomarkers in normal pressure hydrocephalus: evidence for the role of neuroimaging. J. Neurosurg. 110, 837–851. doi: 10.3171/2007.9.17572, PMID: 18991499

[ref47] Tarnaris A. Toma A. K. Kitchen N. D. Watkins L. D. (2009). Ongoing search for diagnostic biomarkers in idiopathic normal pressure hydrocephalus. Biomark Med. 3, 787–805. doi: 10.2217/bmm.09.37, PMID: 20477715

[ref48] Tarnaris A. Watkins L. D. Kitchen N. D. (2006). Biomarkers in chronic adult hydrocephalus. Cerebrospinal Fluid Res. 3:11. doi: 10.1186/1743-8454-3-11, PMID: 17020616 PMC1617118

[ref49] Wang Z. Zhang Y. Hu F. Ding J. Wang X. (2020). Pathogenesis and pathophysiology of idiopathic normal pressure hydrocephalus. CNS Neurosci. Ther. 26, 1230–1240. doi: 10.1111/cns.13526, PMID: 33242372 PMC7702234

[ref50] Wyss-Coray T. (2016). Ageing, neurodegeneration and brain rejuvenation. Nature 539, 180–186. doi: 10.1038/nature20411, PMID: 27830812 PMC5172605

[ref51] Xiang M. Wang Y. Gao Z. Wang J. Chen Q. Sun Z.. (2022). Exploring causal correlations between inflammatory cytokines and systemic lupus erythematosus: a Mendelian randomization. Front. Immunol. 13:985729. doi: 10.3389/fimmu.2022.985729 36741410 PMC9893779

[ref52] Xiao Z. Wang Z. Zhang T. Liu Y. Si M. (2023). Bidirectional Mendelian randomization analysis of the genetic association between primary lung cancer and colorectal cancer. J. Transl. Med. 21:722. doi: 10.1186/s12967-023-04612-7, PMID: 37840123 PMC10577972

[ref53] Yang H. S. Zhang C. Carlyle B. C. Zhen S. Y. Trombetta B. A. Schultz A. P.. (2022). Plasma IL-12/IFN-γ axis predicts cognitive trajectories in cognitively unimpaired older adults. Alzheimers Dement. 18, 645–653. doi: 10.1002/alz.12399, PMID: 34160128 PMC8695625

[ref54] Yeung C. H. C. Schooling C. M. (2021). Systemic inflammatory regulators and risk of Alzheimer’s disease: a bidirectional Mendelian-randomization study. Int. J. Epidemiol. 50, 829–840. doi: 10.1093/ije/dyaa241, PMID: 33313759

[ref55] Zhao J. H. Stacey D. Eriksson N. Macdonald-Dunlop E. Hedman Å. K. Kalnapenkis A.. (2023). Genetics of circulating inflammatory proteins identifies drivers of immune-mediated disease risk and therapeutic targets. Nat. Immunol. 24, 1540–1551. doi: 10.1038/s41590-023-01588-w, PMID: 37563310 PMC10457199

[ref56] Zhong S. Yang W. Zhang Z. Xie Y. Pan L. Ren J.. (2023). Association between viral infections and glioma risk: a two-sample bidirectional Mendelian randomization analysis. BMC Med. 21:487. doi: 10.1186/s12916-023-03142-9 10.1186/s12916-023-03142-9 PMC10698979 38053181

